# Catheter Ablation for Persistent Atrial Fibrillation: Rationale, Evidence, and Contemporary Strategies Beyond Pulmonary Veins

**DOI:** 10.3390/jcm15031167

**Published:** 2026-02-02

**Authors:** Eleonora Ruscio, Mario Marsilia, Gianluigi Bencardino, Maria Lucia Narducci, Francesco Perna, Gianluca Comerci, Gaetano Pinnacchio, Francesco Burzotta, Roberto Scacciavillani, Gemma Pelargonio

**Affiliations:** 1 Department of Cardiovascular Medicine, Fondazione Policlinico Universitario A. Gemelli IRCCS, Largo Agostino Gemelli 8, 00168 Rome, Italy; eleonoraruscio@gmail.com (E.R.); mariomarsilia@gmail.com (M.M.); gianluigi.bencardino@policlinicogemelli.it (G.B.); marialucia.narducci@policlinicogemelli.it (M.L.N.); francesco.perna@policlinicogemelli.it (F.P.); gianluca.comerci@policlinicogemelli.it (G.C.); gaetano.pinnacchio@policlinicogemelli.it (G.P.); francesco.burzotta@policlinicogemelli.it (F.B.); gemma.pelargonio@policlinicogemelli.it (G.P.); 2 Faculty of Medicine and Surgery, Catholic University of the Sacred Heart, 00168 Rome, Italy

**Keywords:** persistent atrial fibrillation, catheter ablation, non-pulmunary vein triggers

## Abstract

Atrial fibrillation (AF) is the most common sustained cardiac arrhythmia and is associated with substantial morbidity and mortality due to increased risks of stroke, heart failure, and death. Catheter ablation is now firmly established as a first-line or early rhythm control strategy in selected patients with paroxysmal AF and as a Class I indication in symptomatic patients with persistent AF refractory to antiarrhythmic drug therapy. However, outcomes remain more variable in persistent and long-standing persistent AF, reflecting greater atrial substrate complexity, procedural challenges, and ongoing uncertainty regarding optimal ablation strategies. This review provides a structured, evidence-based overview of contemporary catheter ablation approaches for AF, with particular emphasis on persistent disease. We discuss the anatomical and mechanistic rationale underlying pulmonary vein isolation and adjunctive lesion sets, summarize current clinical evidence supporting various strategies, and highlight differences in efficacy, safety, and reproducibility. In addition, we address the importance of patient selection, shared decision making, procedural expertise, and comprehensive risk factor modification as integral components of successful long-term rhythm control. By integrating guideline recommendations with mechanistic insights and clinical trial data, this review aims to clarify the current best practices and identify remaining knowledge gaps in the catheter ablation of atrial fibrillation.

## 1. Introduction

Atrial fibrillation (AF) is a supraventricular arrhythmia characterized by disorganized atrial activation and the loss of effective coordinated contraction. It typically originates from automatic or re-entrant ectopic activity at veno-atrial junctions or other arrhythmogenic atrial sites. AF portends the risks of stroke, heart failure, and death through thrombus formation due to atrial stasis and tachycardia-induced ventricular dysfunction. Notably, AF nearly doubles mortality even in individuals without major underlying cardiac disease [1,2]. Therefore, therapeutic strategies primarily aim at restoring and maintaining sinus rhythm to prevent consequences and improve quality of life.

Recent international guidelines, including the 2024 European Society of Cardiology (ESC) and the 2023 ACC/AHA/ACCP/HRS documents, show increasing convergence in their recommendations regarding catheter ablation for AF. Both sets of guidelines endorse catheter ablation as a Class I indication for patients with symptomatic AF in whom antiarrhythmic drugs (AADs) are ineffective, not tolerated, or not desired [1,2]. Importantly, first-line ablation is now endorsed in selected patients with paroxysmal AF (PAF) to improve rhythm control and quality of life. Notably, the ESC guidelines assign a Class I recommendation for first-line ablation in PAF, and the ACC/AHA guidelines designate it as Class IIa, underscoring the importance of shared decision making and operator experience [2].

For persistent AF, ablation carries a Class I recommendation in symptomatic patients that are refractory to at least one AAD [1,2]. As a first-line therapy, it holds a Class IIa indication in carefully selected cases, acknowledging its lower efficacy rates and higher procedural complexity [3]. In long-standing persistent AF, the indication is further downgraded (Class IIb), reserved for highly selected patients, such as those with tachycardia-induced cardiomyopathy [4].

Both European and American guidelines emphasize the need for shared decision making, structured follow-up, and referral to high-volume, experienced centers, particularly for complex ablations or when additional lesion sets are considered [3]. Patient selection should be guided not only by arrhythmia type and duration, but also by atrial substrate characteristics and comorbidity burden, while aligning with realistic expectations [5,6]. In this context, ablation should be regarded as an effective strategy to substantially reduce arrhythmic burden and improve quality of life, acknowledging the potential need for repeat procedures and anticoagulation management [3].

Optimal long-term outcomes rely on the adoption of a holistic approach to AF and to atrial cardiomyopathy in general, which includes aggressive risk factor modification together with an effective catheter ablation procedure [7]. In this regard, the optimal ablation strategy in persistent AF remains an elusive concept and the aim of this review is to provide a structured, evidence-based overview of various ablation approaches, outlining their anatomical rationale, mechanistic purpose, and current clinical support within contemporary practice [8].

The methodological approach adopted for literature identification and selection is described in the Supplementary Materials.

## 2. Ablation Techniques for Atrial Fibrillation: Radiofrequency, Cryoballoon, and Pulsed Field

The ablation energy source significantly influences lesion quality, procedural safety, and long-term efficacy, particularly in achieving the main goal of AF ablation—pulmonary vein isolation (PVI).

The three main modalities currently employed are radiofrequency ablation (RFA), cryoballoon ablation (CBA), and the more recent pulsed field ablation (PFA).

### 2.1. Radiofrequency Ablation

RF ablation employs alternating current to deliver thermal injury and achieve conduction block. It was the first energy source introduced and remains the most extensively studied, supported by robust safety and efficacy data [3]. Randomized and observational studies have shown that RFA reduces AF recurrence compared with AAD therapy [9]. Technological refinements, such as contact force-sensing catheters and high-power, short-duration protocols, have improved lesion durability and procedural efficiency [9,10,11]. Meta-analyses confirm durable pulmonary vein isolation and low complication rates as atrio-esophageal fistula representing a rare (<0.1%) but often fatal complication [12] with contemporary RFA techniques [13].

Pulmonary vein stenosis has been reported with all energy sources—but mostly with RFA, with an incidence approximately of 0.5–1, largely mitigated by antral rather than ostial ablation [14,15].

### 2.2. Cryoballoon Ablation

Cryoenergy induces tissue necrosis by freezing, creating a circumferential lesion at the pulmonary vein ostium. It provides a “single shot” approach with a simplified workflow and reproducible results. Large, randomized trials demonstrated the non-inferiority of CB compared with RF ablation in paroxysmal AF [16,17,18]. Meta-analyses consistently show similar efficacy and safety between CBA and RFA, although transient phrenic nerve palsy occurs more frequently with CB [19,20].

### 2.3. Pulsed Field Ablation

PFA represents a paradigm shift, using non-thermal electroporation to selectively ablate myocardial cells while sparing adjacent structures. Preclinical and early human studies demonstrated myocardial specificity and minimal collateral damage [21]. Recent multicenter randomized trials confirmed the safety and efficacy of PFA in both paroxysmal and persistent AF [22,23].

Systematic reviews and large registries have reported shorter procedural times, high acute success rates, and reduced collateral injury compared with thermal ablation [24,25,26]. Meta-analyses suggest that PFA shortens left atrial dwell time and fluoroscopy exposure while maintaining comparable arrhythmia-free survival [26,27].

However, despite its favorable safety profile, isolated reports of hemolysis and acute kidney injury or transient coronary spasm highlight the need for further long-term safety data [28,29].

### 2.4. Ablation Strategies in Persistent Atrial Fibrillation

In persistent AF, pulmonary vein isolation (PVI) remains the procedural cornerstone but frequently fails to ensure durable rhythm control due to the presence of a more advanced and heterogeneous atrial substrate [4,6,9]. This limitation has prompted the development of adjunctive lesion sets aimed at targeting non-pulmonary vein triggers, interrupting macro-reentrant circuits, and modifying atrial substrate involved in AF maintenance. These strategies and their background will be detailed in the following sections.

### 2.5. Evidence-Based Lesion Sets

#### 2.5.1. Pulmonary Vein Isolation

Pulmonary vein isolation remains the cornerstone of catheter ablation for AF, yet its efficacy is consistently lower in persistent compared with paroxysmal AF. Early studies showed that a single PVI procedure achieves more than 70% arrhythmia-free survival in paroxysmal AF but this drops to nearly twenty percent in persistent AF [30], a difference confirmed by contemporary analyses reporting long-term success rates around 40–45% with PVI alone in non-paroxysmal AF [31]. Even with durable lesion techniques, one-year arrhythmia-free survival in persistent AF seldom exceeds 43–67%, which is a rate markedly below that observed in paroxysmal forms [32]. These findings reflect the more complex atrial substrate typical of persistent AF, where arrhythmia maintenance involves not only pulmonary vein triggers but also extra-PV sources. Within this context, the strategy used to achieve PVI significantly influences outcomes. Wide antral ablation, which encompasses a broader region at the pulmonary vein antra and often part of the posterior wall, has demonstrated superior long-term efficacy compared with ostial ablation, as shown in meta-analyses of RF procedures involving more than a thousand patients [33,34].

#### 2.5.2. Posterior Wall

The posterior wall of the left atrial wall shares a common embryological origin with the pulmonary veins, arising from the embryonic venous plexus, a relationship that contributes to the marked arrhythmogenic predisposition of this region [35]. This anatomic continuity provides the conceptual foundation for posterior wall-directed ablation strategies, which historically evolved from the surgical Cox–Maze procedure, where full-thickness incisions created atrial compartmentalization and prevented macro-reentrant propagation [36]. Replicating such transmurality through catheter ablation, however, remains inherently challenging, particularly along the esophagus-adjacent posterior wall, leading to heterogeneity in lesion durability and clinical outcomes [36]. Evidence regarding adjunctive posterior wall isolation (PWI) in persistent AF remains inconclusive. Early randomized work by Tamborero et al. failed to demonstrate incremental benefit of adding PWI to PVI [37], and the multicenter CAPLA trial subsequently confirmed the absence of an improvement in arrhythmia-free survival, while also reporting longer procedures and a trend toward higher complication rates [38]. Meta-analytic data showed mixed results: Li et al. observed no overall reduction in atrial arrhythmia recurrence, although a subgroup with smaller left atrial dimensions appeared to benefit [39], while other analyses have highlighted the pro-arrhythmic potential of incomplete, non-transmural lines, particularly when roof line conduction gaps persist [40]. More favorable outcomes have been reported in selected cohorts. Bai et al. demonstrated superior long-term freedom from arrhythmia with adjunctive PWI, without an increase in procedural complications, underscoring the potential value of complete bidirectional block in appropriately chosen patients [41]. Consistently, Natale et al. emphasize that divergent trial results may stem from methodological variability, including differences in lesion design (“box lesion” vs. point-by-point technique), operator-dependent transmurality, inconsistent rhythm-monitoring protocols, and the frequent omission of triggers arising from the inferior posterior wall, below the line connecting the inferior pulmonary veins [42]. Anatomical factors are also relevant: the septopulmonary bundle, a subepicardial tract spanning the roof and posterior wall, may maintain epicardial conduction despite continuous endocardial lesions, contributing to roof line gaps. The introduction of PFA has renewed interest in PWI owing to its tissue selectivity, procedural efficiency, and enhanced safety profile. Observational data from Badertscher et al. report a 100% acute PWI success rate, short procedural times, and the absence of major complications, with encouraging early arrhythmia-free rates [43]. Conversely, findings from the large MANIFEST-PF registry do not demonstrate the clinical advantage of adjunctive PWI over PVI alone in persistent AF [25]. As highlighted by Natale et al., these negative results require cautious interpretation given significant limitations, including a small number of PWI procedures, the lack of systematic assessment of lesion durability, substantial inter-center variability, and heterogeneous follow-up strategies [42].

#### 2.5.3. Mitral Isthmus and Vein of Marshall

Achieving durable conduction block across the mitral isthmus (MI) remains one of the most technically challenging components of atrial fibrillation ablation. The lateral or posterior MI, extending from the mitral annulus to the left inferior pulmonary vein, frequently serves as the substrate for peri-mitral macro-reentry; however, endocardial radiofrequency alone often fails to achieve durable bidirectional block, with incomplete lesions reported in up to 40–50% of cases and residual iatrogenic flutter occurring in 10–15% [36]. This limitation arises from the complex epicardial architecture of the isthmus itself, where structures such as the coronary sinus musculature and the vein of Marshall (VOM) create conduction bridges that allow residual epicardial–endocardial breakthrough despite extensive endocardial ablation. As a result, incomplete posterior MI block remains a major driver of recurrent peri-mitral flutter [44].

Within this mechanistic context, the VOM represents a targeted epicardial adjunct designed to overcome the dominant cause of MI block failure. Ethanol infusion into the VOM induces transmural necrosis along the Marshall bundle and adjacent epicardial fibers, zones that are notoriously resistant to radiofrequency energy, thereby facilitating MI block in cases where endocardial ablation alone remains insufficient [45]. The VOM’s embryological origin from the left cardinal vein, and its muscular continuity with both the coronary sinus and the posterior left atrium provide it with the capacity to sustain ectopic triggers, autonomically modulated activity, and peri-mitral reentry circuits, contributing to the initiation and maintenance of AF [46].

Clinical evidence strongly supports this adjunctive approach. The VENUS randomized trial demonstrated an 11% absolute reduction in AF recurrence at one year when VOM ethanol infusion was added to PVI [47]. The MARSHAL-Plan study expanded this concept by combining PVI, VOM ethanolization, and targeted linear lesions (mitral isthmus, left atrial dome, and cavotricuspid isthmus), achieving superior sinus rhythm maintenance at 12 months compared with PVI alone [48]. A comprehensive meta-analysis of more than one thousand three hundred patients confirmed that adding VOM ethanol infusion improves arrhythmia-free survival and increases the rates of durable MI block without elevating major complication risk [49].

Despite its advantages, VOM alcoholization remains technically demanding, with procedure complexity and anatomical variability contributing to failures in VOM cannulation; complications, although uncommon, include phrenic nerve injury, pericarditis, and coronary sinus trauma [45].

The anterior (superior) MI represents an anatomically distinct alternative when posterior MI block remains elusive. This line connects the mitral annulus to the right superior pulmonary vein and courses through a thicker region of the anterior left atrium, largely free of epicardial connections, thereby allowing more predictable transmural lesion formation. Clinical studies have demonstrated high rates of flutter termination and durable block using the superior line [50]. In a recent study in patients with persistent AF and anterior scar, the anterior mitral line, on top of standard treatment, demonstrated improved long-term outcomes, highlighting its usefulness not only for the termination of atrial tachycardias/flutters but also as a debulking target [51]. Recently, PFA has been applied successfully to this region, enabling the rapid creation of anterior MI lesions with high acute success and encouraging mid-term arrhythmia control [52].

#### 2.5.4. Non-Pulmonary Vein Triggers

Several adjunctive atrial lesion sets have been explored to address non-pulmonary vein substrates sustaining AF, particularly in persistent or recurrent forms.

In this setting, a systematic search for non-pulmonary vein triggers should be performed after the confirmation of durable PVI using standardized provocative protocols. High-dose isoproterenol infusion (20–30 μg/min for ≥10 min), performed in sinus rhythm with multipolar catheter mapping of both atria, allows identification of extra-PV triggers [53].

The left atrial appendage (LAA) represents both a frequent non-PV trigger source and a structure prone to functional isolation during extensive linear ablation; intentional LAA isolation has been associated with improved rhythm control but raises concerns regarding impaired appendage function and thromboembolic risk, prompting the consideration of percutaneous closure in selected cases [54,55,56]. In this context, percutaneous left atrial appendage occlusion with dedicated devices (e.g., Watchman and Amulet) represents a valuable adjunct in selected patients undergoing LAA isolation or with contraindications to long-term anticoagulation, with growing evidence supporting its safety and efficacy [56]. Ongoing device development may further expand its role in integrated AF management. Epicardial structures such as the coronary sinus (CS) contribute additional non-PV triggers and reentrant pathways. In fact, CS-related triggers are frequently revealed during ablation through provocative maneuvers such as isoproterenol infusion or burst pacing [55]. Achieving durable CS isolation is challenging, and VOM ethanol infusion, as previously discussed, serves as an effective adjunct by eliminating epicardial connections that sustain residual conduction despite extensive endocardial ablation [45]. PFA has also been evaluated for direct CS isolation, with La Fazia et al. reporting a 62% acute success rate and excellent safety, but durable isolation was successful in approximately one percent at a three-month remapping, underscoring its current limitations for stable CS lesion formation [57].

On the right atrial side, several structures represent clinically relevant non-PV sources of atrial ectopy and focal or reentrant firing. The superior vena cava (SVC) is among the most frequent, with myocardial sleeves capable of rapid focal discharges; up to 12% of non-PV triggers originate from this region, and targeted isolation, guided by detailed mapping and phrenic nerve pacing, has been associated with reduced recurrence in selected patients [58,59,60]. The crista terminalis (CT) is another major substrate, characterized by bundles capable of automatic or triggered activity. Early mechanistic studies demonstrated that CT-origin tachycardias exhibit sharp local electrograms, centrifugal activation patterns, and consistent localization to the mid-to-upper CT, with high acute success achievable through precise activation mapping [61,62]. More recent work confirms stable long-term outcomes when the earliest activation point is carefully defined and phrenic nerve mapping is performed to avoid diaphragmatic injury [63]. The Eustachian ridge (ER), the inferior extension of the CT, can also participate in arrhythmogenesis: it forms a functional boundary of the cavotricuspid isthmus (CTI) and may occasionally host focal non-PV triggers, as shown in reports of AF arising from a prominent ER successfully ablated with high-density mapping guidance [64,65,66]. Although CTI ablation remains the primary strategy for modifying this region, emerging data suggests that PFA may offer a safe, non-thermal option for achieving bidirectional CTI block, though dedicated evidence for ER-specific arrhythmias remains limited [67,68].

#### 2.5.5. Complex Fractionated Atrial Electrograms

Complex fractionated atrial electrograms (CFAE) initially proposed as surrogate markers of AF-maintaining sites [9,69], were hypothesized to reflect zones of slowed conduction, pivoting wavefronts, or autonomic modulation within a remodeled substrate.

Although early studies suggested incremental benefit over PVI, subsequent randomized evidence has not confirmed this premise [70]. In STAR-AF II, adding CFAE ablation to PVI in persistent AF failed to improve arrhythmia-free survival while prolonging procedure duration [9]. Similarly, rotor- or driver-guided strategies, including FIRM and contemporary phase mapping approaches, did not demonstrate superiority over conventional ablation in redo or non-paroxysmal AF cohorts [71,72]. High-density mapping studies have since shown that many CFAE regions represent passive bystander activity, particularly along the septum and posterior wall, rather than true AF drivers [73]. Recent reviews emphasize substantial methodological heterogeneity, limited reproducibility, and the tendency of mapping systems to identify conduction phenomena rather than stable driver sources [74,75].

Another novel approach that follows in these footsteps employed Artificial Intelligence-guided ablation targeting spatio-temporal electrogram dispersion in addition to PVI, showing superiority to PVI alone in terms of achieving freedom from AF at 1 year [76].

Consistent with emerging mechanistic understanding, current guidelines consider CFAE, or driver-guided ablation, to be investigational and recommend its use only in highly selected cases within experienced centers [1,2].

#### 2.5.6. Low-Voltage Substrate-Guided Ablation

Beyond electrogram- or trigger-based approaches, atrial substrate modification targeting low-voltage myocardium has emerged as a mechanistically grounded strategy in persistent AF. Low bipolar voltage areas identified during sinus rhythm are considered markers of advanced structural remodeling and re-entrant vulnerability rather than epiphenomena of fibrillatory conduction [77]. In the multicentre randomized ERASE-AF trial, the individualized ablation of low-voltage atrial myocardium in addition to PVI significantly reduced atrial arrhythmia recurrence compared with PVI alone, without a clinically relevant increase in procedural complications [78].

In contrast, the randomized SUPPRESS-AF trial did not demonstrate a significant overall benefit of empiric low-voltage area ablation added to PVI [79]. However, a prespecified subanalysis showed that the efficacy of low-voltage-guided ablation was significantly enhanced in patients with advanced left atrial enlargement, suggesting that voltage-defined substrate modification may be particularly relevant in more remodeled atria, where arrhythmia maintenance is predominantly substrate-driven rather than trigger-dependent [80].

More recently, non-thermal energy sources have been applied to substrate-guided strategies. In a multicenter experience, pulsed field ablation (PFA) enabled the efficient targeting of the atrial substrate with a favorable procedural safety profile, supporting the technical applicability of low-voltage-guided ablation using emerging energy modalities [81].

While these data represent the strongest randomized evidence supporting substrate-guided ablation in persistent AF, broader validation and further standardization of voltage mapping protocols remain necessary.

#### 2.5.7. Autonomic Modulation Strategies: Ganglionated Plexi and Renal Denervation

Adjunctive autonomic modulation approaches, including the ablation of ganglionated plexi (GPs) and renal denervation (RDN), have been explored to enhance outcomes in AF. Experimental and early clinical observations linked heightened vagal responses during the high-frequency stimulation of left-atrial GPs with AF initiation and persistence, supporting the rationale for autonomic substrate modification [82]; however, in the randomized GANGLIA-AF trial, GPs ablation alone failed to outperform PVI in preventing atrial arrhythmias, while several GPs remain difficult to access with an endocardial approach, limiting procedural reproducibility [83]. Notably, only patients with paroxysmal AF were included. More recently, experimental and clinical data suggest that neuromodulatory effects may occur even without direct GP targeting. In particular, RF energy delivered near autonomic structures can attenuate vagal reflexes in a graded manner, depending on lesion proximity rather than intentional GP ablation [84].

Renal denervation has been investigated as a complementary strategy aimed at reducing cardiac sympathetic drive. In the randomized ERADICATE-AF trial, adding RDN to catheter ablation in patients with paroxysmal AF and hypertension significantly increased 12-month freedom rates from AF, consistent with preclinical data demonstrating reduced fibrosis, improved electrophysiologic homogeneity, and diminished stellate ganglion activity [85]. Nevertheless, results have been inconsistent: in the RDN + AF trial, enrolling a broader, hypertensive population that included approximately forty percent of patients with persistent AF, the addition of RDN failed to confer clinical benefit, leaving unresolved questions regarding the optimal timing, durability of denervation, and patient selection [86].

#### 2.5.8. Redo Ablation and Mapping-Targeted Strategies

Redo procedures following PVI primarily address the reconnection of previously isolated veins; however, contemporary technologies have reduced the frequency of PV reconnection, and a substantial proportion of patients now experience recurrence despite durable PVI, highlighting the growing relevance of extra-PV targets in repeat procedures. In these settings, non-PV triggers, previously discussed, often sustain arrhythmia, and several studies demonstrate that ablating such triggers significantly improves long-term outcomes, particularly in patients with multiple prior failed ablations or long-standing persistent AF [42].

Mapping-targeted ablation strategies yielded inconsistent results. Early phase mapping approaches failed to demonstrate clinical superiority in randomized evaluation, whereas more recent systems have reported encouraging but still preliminary results, often in small or uncontrolled cohorts [71,87,88].

Redo procedures performed with PFA demonstrated high acute success in re-isolating reconnected pulmonary veins and targeting residual arrhythmogenic substrate, with short procedural times and a favorable safety profile. Their long-term durability and incremental benefit over conventional energy sources require further validation [89].

#### 2.5.9. Hybrid Endocardial–Epicardial Ablation

Hybrid endocardial–epicardial ablation has emerged as a mechanistically complementary strategy for persistent and long-standing persistent AF, particularly in patients in whom advanced atrial remodeling limits the efficacy of endocardial ablation alone [4]. Early surgical experience demonstrated the feasibility of minimally invasive epicardial lesion delivery and posterior wall modification, offering more consistent transmurality than endocardial techniques [90]. Electrophysiological endo-epi mapping subsequently revealed that critical arrhythmogenic substrates, including epicardial low-voltage areas and conduction channels, may be exclusively epicardial, supporting the rationale for combined approaches [91]. Contemporary hybrid strategies pair the thoracoscopic epicardial pulmonary vein and PWI with left atrial appendage exclusion, followed by delayed endocardial mapping to confirm bidirectional block and eliminate residual gaps. Randomized evidence has established clear superiority over endocardial ablation alone: in CEASE-AF, hybrid ablation achieved significantly higher arrhythmia-free survival rates at 12 months vs. catheter ablation (71.6% vs. 39.2%; *p* < 0.001), with comparable major complication rates [92]. The CONVERGE trial similarly demonstrated improved rhythm control with hybrid convergent ablation [93] and, more recently, the DEEP multicenter investigational trial, also enrolling prior ablation non-responders, reported a 71.8% arrhythmia-free survival rate at 12 months with an acceptable serious adverse event rate of 6.7% [94].

The VoM represents a distinct epicardial target and a selective adjunct for overcoming MI block failure; its role and technical application have been detailed in the corresponding section above. 

Adjunctive lesion sets are summarized in “Table 1”.

## 3. Discussion

In persistent AF, PVI remains the procedural cornerstone; however, its effectiveness relies not only on durable pulmonary vein disconnection but also on lesion set geometry, with wide antral isolation providing superior substrate modification compared with ostial approaches [30,31,33,34].

Adjunctive strategies seek to address the complex atrial remodeling typical of worse subtypes of AF, yet their incremental clinical value varies considerably [5,9]. For the LA posterior wall, PFA has renewed interest in PWI by enabling efficient, safe lesion delivery. Nonetheless, despite high acute success, the added clinical benefit of PWI over PVI alone remains uncertain: while small PFA series suggest very low recurrence rates [43], larger registries show no significant advantage [25]. Randomized trials specifically evaluating PFA-derived PWI in persistent AF are therefore needed [42].

Moreover, emerging evidence from hybrid endocardial–epicardial strategies further suggests that epicardial access may overcome the anatomical barriers that frequently limit durable endocardial posterior wall isolation. In this regard, the CONVERGE trial demonstrated improved arrhythmia-free survival when posterior wall ablation was performed using a combined hybrid endocardial–epicardial approach, supporting the mechanistic relevance of epicardial substrate modification in this region [93].

In contrast, the mitral isthmus–vein of Marshall (MI–VOM) complex represents a well-defined anatomical–electrophysiological substrate in which epicardial connections, particularly the Marshall bundle, constitute the primary obstacle to durable mitral isthmus block [46]. Ethanol infusion into the vein of Marshall serves as a selective epicardial adjunct that improves lesion completeness, facilitates mitral isthmus block, and enhances outcomes in appropriately selected patients with persistent AF [45,47,48,49].

Other non-pulmonary vein triggers further underscore the heterogeneity of atrial arrhythmogenesis and support a selective, physiology-driven rather than empirical ablation strategy [54,55]. Similarly, complex fractionated atrial electrogram (CFAE) and driver-guided approaches remain investigational, limited by inconsistent mechanistic specificity, poor reproducibility, and lack of standardized mapping methodologies [9,71,87,88].

Autonomic modulation strategies have likewise shown insufficient benefit in persistent AF. Contemporary evidence indicates that advanced AF is dominated by diffuse fibrosis, structural remodeling, and conduction slowing, rendering autonomic influences secondary and explaining the lack of consistent efficacy signals in these populations [83,85,86]. Finally, mapping-guided strategies in redo ablation show promise but remain constrained by variable spatial resolution, inconsistent driver stability, and substantial operator dependence. Their use should therefore be limited to investigational settings until validated in large, standardized, randomized trials [42,71,72].

After catheter ablation, particularly in persistent AF, where early recurrences are common and often reflect transient inflammation and incomplete lesion maturation, short-term antiarrhythmic drug therapy is frequently used during the early post-procedural (“blanking”) phase. Randomized trials indicate that this strategy reduces early atrial tachyarrhythmias- and arrhythmia-related hospitalization without conferring durable long-term rhythm control. In the 5A Study and AMIO-CAT, short-term antiarrhythmic drug therapy did not significantly improve the 6-month arrhythmia-free survival rate but consistently reduced early symptomatic recurrences, cardioversion, and hospitalization [95,96]. Accordingly, contemporary guidelines frame post-ablation AAD use as an individualized, time-limited strategy, with discontinuation generally considered once rhythm stability is achieved [1,4]. Real-world registry data further demonstrate its wide variability in practice and limited long-term benefits, supporting its selective use focused on early symptom control rather than sustained disease modification [97,98].

## 4. Conclusions

Overall, cumulative evidence indicates that successful catheter ablation in persistent AF requires durable PVI integrated with selective, mechanism-based adjunctive strategies rather than broad empirical lesion sets. Low-voltage substrate-guided ablation represents a mechanistically grounded approach to personalization in persistent AF, targeting advanced atrial remodeling beyond pulmonary vein triggers. The available randomized data support its selective integration with PVI to enhance long-term rhythm control.

Future progress in this field will depend on substrate-guided personalization, the rigorous evaluation of PFA-enabled strategies, and the improved standardization of contemporary mapping technologies. Finally, hybrid endocardial–epicardial ablation currently offers the most comprehensive substrate modification strategy, particularly for patients with extensive atrial remodeling or prior ablation failure. Given its procedural complexity and the need for coordinated surgical–electrophysiological workflows, its use should remain confined to high-expertise multidisciplinary centers.

**Table 1:** Summary of adjunctive lesion sets beyond PVI in persistent AF.

Lesion Set/Section	Indications	Safety	Evidence Summary	Clinical Role
**Posterior Wall (Roof/Floor or “box”)**	Persistent AF with posterior wall vulnerability or low voltage.	Thermal: esophageal injury risk; PFA: low acute complications and rare coronary spasm	RCTs negative [37,38]; benefit only in selected anatomies [41]; and PFA promising but registry data neutral [44].	Selective
**Mitral Isthmus (Lateral/Anterior)**	Peri-mitral flutter; macro-reentrant circuits.	RF: CS injury risk; PFA: low acute complications	Endocardial block often incomplete; anterior line more reliable; and PFA with favorable acute success [52,99].	Selective
**Vein of Marshall**	Adjunct to MI block; peri-mitral circuits; and selected persistent AF.	Rare CS trauma, pericarditis, and phrenic nerve injury; overall low major complications.	VENUS and MARSHAL-Plan positive; improved MI block durability [47,48].	Essential in selected cases
**Left Atrial Appendage**	Redo AF; LAA triggers; and maze-like “unintentional” isolation.	Risk of impaired flow and thrombus; may need LAA occlusion.	Improves rhythm control but increases thromboembolism; LAAO often required [54,56].	Selective–high expertise
**Coronary Sinus**	CS triggers; epicardial MI/peri-mitral flutter contribution.	RF: perforation risk; PFA safe but limited durability; and phrenic nerve caution.	Durable isolation difficult; VOM facilitates; and PFA high acute and low durable (≈1%) [55,57].	Selective
**Right-Atrial Triggers (SVC, CT, Eustachian Ridge)**	Documented SVC firing; CT/ER focal AT/AF; and CTI flutter.	SVC: phrenic palsy risk; CT: low complications; and PFA safe	SVC: 6–12% triggers; CT high acute success; and ER mostly CTI component [63,66].	Selective; essential only for CTI
**CFAE**	Persistent AF substrate modulation.	Prolonged procedures increase thermal injury.	STAR-AF II negative; rotor/phase mapping inconsistent [9].	Investigational
**LOW VOLTAGE AREA**	Persistent AF with advanced atrial remodeling.	Increased ablation burden and longer procedures; thermal injury risk proportional to lesion extent; and PFA safe.	ERASE-AF positive randomized trial [78]; SUPPRESS-AF neutral overall with efficacy signal in patients with advanced left atrial enlargement [79,80]; and feasibility of PFA-based low-voltage targeting demonstrated, but long-term benefit unproven [81].	Selective.
**Autonomic Modulation (GPs, RDN)**	Vagal AF (GPs); hypertension + AF (RDN).	GPs: vagal injury risk; RDN: vascular access risks.	GPs: no benefit; RDN: paroxysmal AF positive and persistent AF mixed/negative [83,86].	Investigational
**Hybrid Endo–Epicardial Ablation**	Persistent/long-standing AF with advanced substrate or prior failed ablation.	Surgical access; low esophageal risk; and acceptable complication rate.	RCTs (CONVERGE, CEASE-AF) show superior arrhythmia-free survival vs. endocardial ablation alone, with improved lesion durability through direct treatment of epicardial substrates not accessible endocardially [92,93].	Selective, for experienced centers

Abbreviations. MI: mitral isthmus, AT: atrial tachycardia, CFAE: complex fractionated atrial electrograms, CS: coronary sinus, CT: crista terminalis, CTI: cavotricuspid isthmus, ER: Eustachian ridge, GPs: ganglionated plexi, LAA: left atrial appendage, LAAO: left atrial appendage occlusion, RDN: renal denervation, SVC: superior vena cava; and VOM: vein of Marshall.

## Data Availability

This study did not generate or analyze new datasets. All information discussed is derived from previously published studies, which are cited in the reference list.

## Supplementary Materials

The following supporting information can be downloaded at https://www.mdpi.com/article/10.3390/jcm15031167/s1. Figure S1: Schematic representation of the structured literature identification and selection process adopted for this narrative review.
