# Supplementary material for: Catheter Ablation for Persistent Atrial Fibrillation: Rationale, Evidence, and Contemporary Strategies Beyond Pulmonary Veins
Source: J Clin Med. 2026 Feb 2;15(3):1167. doi: 10.3390/jcm15031167 (PMC12898471; doi:10.3390/jcm15031167)

Supplementary Materials. Literature Identification and Selection Process

This narrative review was conducted according to established methodological principles for narrative reviews, aiming to provide a comprehensive and critical synthesis of contemporary catheter ablation strategies for persistent atrial fibrillation. The literature search was performed using the PubMed/MEDLINE database and included articles published up to December 2024.
The search strategy employed combinations of relevant keywords and Medical Subject Headings (MeSH), including "atrial fibrillation," "persistent atrial fibrillation," "catheter ablation," "pulmonary vein isolation," as well as specific terms related to adjunctive ablation strategies and energy sources. To enhance identification of clinically and conceptually relevant literature, reference lists of key publications and international guideline documents were also examined manually.
Consistent with established methodological guidance for narrative reviews, including the SANRA framework(1), the search was not designed to be exhaustive nor to follow the formal reporting requirements of systematic reviews, such as fully reproducible search strategies, predefined inclusion or exclusion criteria, or mandatory flow diagrams as required by PRISMA recommendations for systematic reviews. Instead, priority was given to international consensus guidelines, randomized controlled trials, meta-analyses, and high-quality observational studies considered most representative of contemporary clinical practice and emerging evidence. Article selection was guided by relevance to the narrative scope and conceptual contribution rather than by formal eligibility thresholds.
No formal quality scoring, standardized data extraction forms, or quantitative synthesis were undertaken, in keeping with the narrative review design. To improve transparency of the literature identification and selection process while preserving the narrative nature of the review, a schematic selection flow-chart is provided in the Supplementary Material.

1. Baethge C, Goldbeck-Wood S, Mertens S. SANRA—a scale for the quality assessment of narrative review articles. Res Integr Peer Rev. 2019;4(1).

Figure S1. Schematic representation of the structured literature identification and selection process adopted for this narrative review.

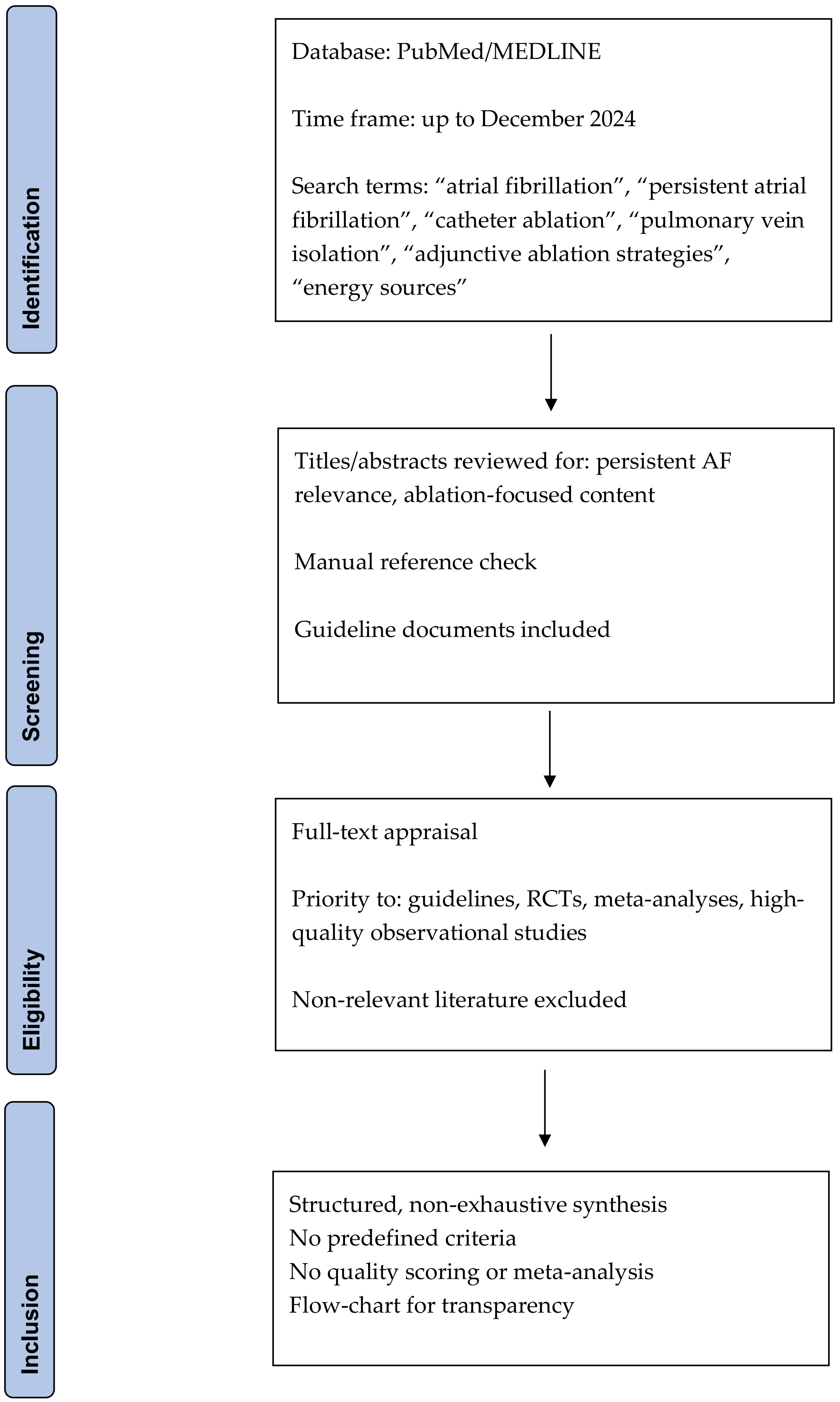

Supplement: Supplementary file 1 [file jcm-15-01167-s001.zip › jcm-4107951-supplementary.pdf]
